# Substance consumption in adolescents with and without an immigration background: a representative study—What part of an immigration background is protective against binge drinking?

**DOI:** 10.1186/s12889-016-3796-0

**Published:** 2016-11-14

**Authors:** Carolin Donath, Dirk Baier, Elmar Graessel, Thomas Hillemacher

**Affiliations:** 1 Center for Health Services Research in Medicine, Department of Psychiatry and Psychotherapy, Friedrich-Alexander-University Erlangen-Nuremberg/University Clinic Erlangen, Schwabachanlage 6, 91054 Erlangen, Germany; 2 Criminological Research Institute of Lower Saxony, Lützerodestr. 9, 30161 Hannover, Germany; 3 Center for Addiction Research, Clinic for Psychiatry, Social Psychiatry and Psychotherapy, Hannover Medical School, Carl-Neuberg-Str. 1, 30625 Hannover, Germany

**Keywords:** Alcohol drinking, Tobacco use, Cannabis, Adolescent, Transients and immigrants, Acculturation, Binge drinking, Risk factors, Protective factors, Attitude

## Abstract

**Background:**

Representative data indicate that adolescents with an immigration background show less harmful patterns of consumption, for example, they practice binge drinking less often. It remains to be shown whether this also applies to substances such as tobacco and cannabis and if the “healthier” patterns of consumption are permanent or if they gradually disappear as the level of integration increases. Using representative data, the current study was designed to a) present the epidemiology of the consumption of alcohol, tobacco, and cannabis of adolescents with and without an immigration background in 2013 and b) to analyze which immigration-specific variables predict problematic alcohol consumption in adolescents with an immigration background.

**Methods:**

A representative, written survey was administered to 9512 students in the 9th grade from Lower Saxony, Germany in 2013 by the “Kriminologisches Forschungsinstitut Niedersachsen (KfN).” Data were collected from 1763 adolescents with an immigration background regarding their cultural, structural, social, and identificative integration. These variables were introduced as predictors in a multiple logistic regression analysis with binge drinking during the last 30 days as the dependent variable.

**Results:**

Compared with German adolescents without an immigration background, significantly fewer adolescents with an immigration background had already tried alcohol, but they were significantly more likely to report experience with cigarettes and cannabis. In the group of adolescents with an immigration background, the percentage of binge drinkers fluctuated by country of origin (*p* < .001). In the regression model, binge drinking was associated with a lower targeted school leaving certificate (*p* < .001), not living on social welfare (*p* = .038), and the strong assimilation (*p* = .015) of the adolescent. Binge drinking was negatively associated with attitudes that favored segregation (*p* < .001) and a stronger attachment of the parents to the traditions of their country of origin (*p* = .003).

**Conclusions:**

It cannot be confirmed that adolescents with an immigration background generally show less harmful patterns of consumption. Distinctions have to be made regarding the substance, the adolescent’s country of origin, and the level of assimilation or segregation of the adolescent and his/her family.

**Electronic supplementary material:**

The online version of this article (doi:10.1186/s12889-016-3796-0) contains supplementary material, which is available to authorized users.

## Background

Acculturation is known as the process of culture change and adaptation that occurs when individuals with different cultures come into contact [1].

Esser [2, 3] claims that the extent of acculturation can be described by four categories of integration (synonymously used for acculturation): cultural integration, structural integration, social integration, and identificative integration. Cultural integration describes a process of cognitive socialization, or more specifically, how immigrants learn typical cultural rules and skills, particularly at the linguistic level. Structural integration is known as placing the immigrant in the social system (e.g. in a particular social position). The term social integration means that people are socially part of the destination country, but at the same time, maintain bonds with their country of origin. Identificative integration is the last step of integration, and it describes the immigrant’s attitude toward him-/herself as part of the social system. It is an emotional bonding with his/her environment and leads to the subjective feelings of unity and national pride.

It is known that during the acculturation process, the health status of immigrants adapts to the population of the immigration country. These effects are especially apparent the longer the immigrant lives in the destination country, for example, in the second generation (e.g. [4, 5]). This has been shown for cholesterol levels [5] and BMI [6] in immigrants who formerly immigrated to the U.S., presumably due to the acculturation of lifestyle (diet, exercise patterns).

Furthermore, sociological parameters also seem to adapt, for example, the age at which mothers give birth to their first child. Immigration to Germany seems to postpone offspring. While women are 19.9 years old on average when they give birth to their first child in Turkey, Turkish women who immigrated to Germany tend to be 23.3 years old when they have their first child. Women with a Turkish immigration background who belong to the second generation of immigrants tend to be slightly older at 23.7 years. At the time of this assessment, the age at which native German women have their first child was reported to be 27.8 years [7].

Thus, it has to be expected that acculturation also affects substance consumption. Several recent representative studies have described a “less harmful” consumption pattern, but most have concentrated on alcohol consumption (e.g. [8, 9]). For example, according to the German Federal Center for Health Promotion (BZgA), the rate at which older adolescents reported engaging in binge drinking at least once in the last 4 weeks was 47.7% for native German adolescents and varied from 16.0 to 36.1% by country of origin for adolescents with immigration backgrounds. Research has yet to determine whether such favourable consumption patterns also apply to other substances such as tobacco and cannabis and whether the effects reported for alcohol are stable and can be replicated in other representative data sets. There is also a need to uncover whether the advantageous (alcohol) consumption patterns slowly disappear as acculturation to the German wet drinking culture occurs and as adolescents assimilate more to the cultures of their immigration countries such as the acculturation processes from other health parameters would suggest.

Using a representative sample of adolescents, the current study was designed a) to present up-to-date epidemiological data on alcohol, tobacco, and cannabis consumption in adolescents with and without an immigration background and b) to examine which immigration-specific predictors are associated with health-related problematic alcohol consumption behavior (binge drinking) in adolescents with an immigration background.

### Research Questions

Thus, we aimed to answer two research questions in this article:

1. How prevalent is alcohol, tobacco, and cannabis consumption as well as binge drinking in a representative sample of 9th graders in Germany in 2013 in general and with respect to their immigration background?
2. Which specific immigration-associated variables predict binge drinking in the subgroup of adolescents with an immigration background?

## Methods

### Study Design, Data Collection, Ethical Considerations

The goal was to conduct a representative survey of the whole of Lower Saxony, Germany for adolescents in the 9th grade, reaching a total of 10,000 students (population in the school year 2012/2013: 90,852 students). All school forms were to be considered except for special-needs schools whose focus differed from the focus on learning (e.g. mental or physical handicaps). To interview 10,000 students, a sample of about 460 school classes is necessary. As experience from previous studies has shown that about three out of every ten classes that are contacted do not take part in the survey, the number of classes to be included was increased accordingly. It was possible to calculate how many classes had to be included in the sample for each school type from their share of the population. A random selection of classes stratified by school type was necessary as the average class size varied greatly between the different school types: On average, there are 11 students in the classes from the special-needs schools, but there are 26 students in the German academic high school (“Gymnasium”) classes.

**Fig. 1:**
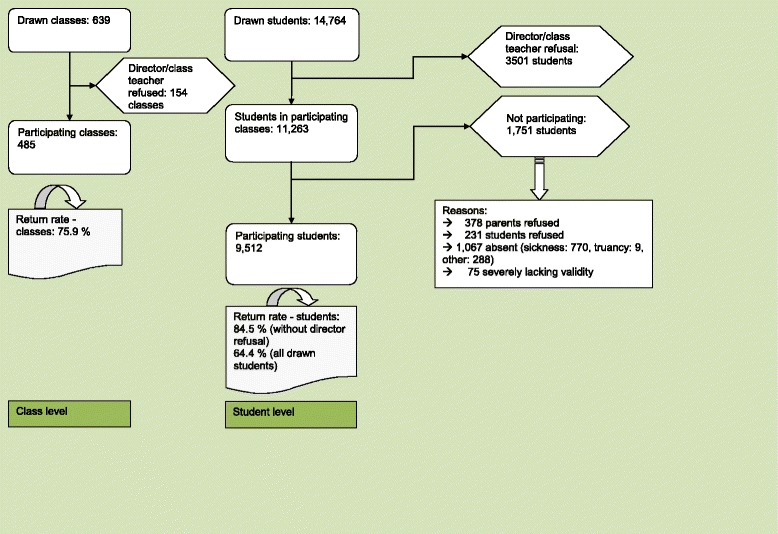
Sample flow-chart

In Lower Saxony, there are a total of seven different school types. Within each school type, school classes were drawn randomly, totalling 639. Both state schools and privately operated schools were included in the survey. Out of the selected classes, 154 did not participate, and the surveys were administered in 485 classes. In the end, a total of 9512 adolescents were reached in the participating classes. This equaled a return rate of 64.4%. The sample acquisition process and number/reasons for refusal are depicted in Fig. 1.

The research project was implemented by the Lower Saxony State Ministry of Science and Culture. The survey was ethically audited and approved by the ethical commission of the Ministry of Education of Lower Saxony. Consequently, the survey was strictly anonymized—no names, no addresses, and no school addresses were obtained. Written consent was obtained from the parents of the adolescents. A one-page information letter was distributed some days before the survey was to take place to inform the students’ parents about the survey. If the parent(s) did not give their consent, the student could not participate in the survey. Furthermore, the students were themselves free to decide whether they wanted to take part in the survey. If they did not wish to participate, they were given alternative material by their teachers and were not discriminated against in any way.

The survey itself was administered in class, usually in the presence of a teacher or another adult supervisor. The study assistants introduced themselves briefly to the students at the beginning of the class and handed out the questionnaires. Afterwards, the study assistants read the first page of the questionnaire aloud. In addition to other information, the first page explained the anonymous and voluntary nature of the survey. The students then worked on the questionnaire collectively up to page 6, i.e. the study assistants read the questions and the corresponding choices aloud and gave further instructions or information if necessary. From page 6 onwards, the students could then fill in the total of 34 pages of the questionnaire on their own. The procedure was different only in the special-needs schools where the entire total of 21 pages of the questionnaire was put on an overhead transparency and read aloud to make it possible for students with reading deficiencies to participate. On average, the survey lasted 92 min. At the end of the survey, the questionnaires were collected and put into an envelope, which was then closed and sealed. The surveys were administered between January 07, 2013 and May 05, 2013.

### Sample

The sample was representative for one German state but not for all of Germany. Regarding the composition of school types, the realized sample corresponded rather well to the composition of the population. For example, in the 2012–2013 school year, 7.5% of all students went to a secondary general school (9 years). In the sample, the percentage of students in secondary general schools was also 7.5%. The students from the special-needs schools showed the highest relative deviation: In the population, there are 1.2 times more special-needs students than in the sample (3.2% compared to 2.6%). The second largest deviation could be noted for the comprehensive schools (14.4% in the population compared with 12.7% in the sample). Data weighting was applied to level out such differences. All of the following results are based on weighted data.

The sample characteristics are shown in Table 1. We also collected the following further information about the sample: Besides living with both biological parents, the most common family constellations involved living with the mother and stepfather (10.3%) or living with only the mother (10.9%). Furthermore, 80.3% of the adolescents reported living with siblings. Of these, the largest proportion lived with one sibling (*N* = 4500; 47.3% of the total sample), about one fifth of the whole sample lived with two siblings (*N* = 1811; 19.0%), whereas the remaining lived within larger family structures.

**Table 1:** Sample description (*N* = 9512)

Variable	Frequency (*n*)	% resp. Mean (SD)	Missing *n* (%)
Age	-	14.88 (.74)	17 (0.2 %)
Sex (female)	4677	49.3 %	21 (0.2 %)
Planned type of school leaving certificate			304 (3.2 %)
Secondary general school certificate (9 years)	744	8.2 %	
Secondary modern school certificate (10 years)	4443	48.9 %	
High school diploma (at least 12 years)	3895	42.9 %	
Immigration background (yes)	2277	24.3 %	158 (1.7 %)
Urban/Rural Living:			
Rural (below 10,000 inhabitants)	2635	27.7 %	0 (0 %)
Small Town (below 20,000 inhabitants)	2335	24.5 %	
Urban (below 50,000 inhabitants)	2491	26.2 %	
Metropolitan (over 50,000 inhabitants)	2051	21.6 %	
Living with both corporal parents (yes)	6587	69.7 %	62 (0.6 %)
Living with siblings (no)	1871	19.7 %	262 (2.7 %)
Living on social welfare (yes)	622	6.5 %	132 (1.4 %)

About a quarter (24.3%) of the 9th graders had an immigration background, even though 98% of them were born in Germany. The percentage of people with an immigration background living in the population of Germany is currently 20.3% (16.4 million) [10]. The 24.3% in our sample were not extremely different from the general population. The slightly higher percentage of about 4% more than the general average could be explained by the small age corridor in our sample. The 20.3% of the general population is the average across all age groups, and in Germany, the immigration background rate declines as the age of the population increases. In the 15–20-year age group, in the general population of Germany, the percentage of people with an immigration background is about ¼, which is fairly close to the percentage in our sample [10].

The largest group of immigrants in our sample consisted of adolescents who came from the countries of the former Soviet Union (7.1%). The second largest group of immigrants consisted of Turkish participants (4.5%), and the third largest included Polish (2.8%) participants. The parameters describing the sample are depicted in Table 1.

### Instruments

Substance consumption was investigated by administering substance- (and beverage-) specific items from a representative survey from the Criminological Research Institute of Lower Saxony in 2001 [11] and 2008 [9]. In the current study, only data concerning alcohol, tobacco, and cannabis were analyzed.

The lifetime prevalence of alcohol, tobacco, and cannabis use was assessed with the items “Have you ever drank … (beverage)? /Have you ever smoked cigarettes? /Have you ever tried cannabis/marihuana/pot?” The age at first consumption was equivalently assessed by the question “How old were you when you did this for the first time?” The 12-month prevalence rates for the substances were assessed with “How frequently in the last 12 months did you…?” (… drink (beverage)/smoke cigarettes/try cannabis/marihuana/pot) with the answer formats “never/1 or 2 times/3 to 12 times/several times per month/once a week/several times per week/daily.” For data analysis, the categories were collapsed into the five categories. The item for assessing heavy episodic drinking (binge drinking) was derived from the representative survey of adolescents from the German Federal Center for Health Education [12]. Binge drinking is defined as the consumption of five or more standard drinks during one drinking opportunity. The adolescents were asked a) if they had consumed alcohol in the last 30 days (30-day prevalence) and if yes, b) on how many days they had consumed five or more standard alcoholic drinks in a row. This measure of heavy episodic drinking was used exactly like this in the European representative ESPAD study [13], which had the same age distribution as the current sample.

In line with the standard for German sociodemographic surveys, immigration background was defined as: having at least one parent who was born outside of Germany, having been born outside of Germany oneself, having non-German citizenship, or having at least one parent with non-German citizenship. This method differs from those used in other studies, particularly in the US.

**Fig. 2:**
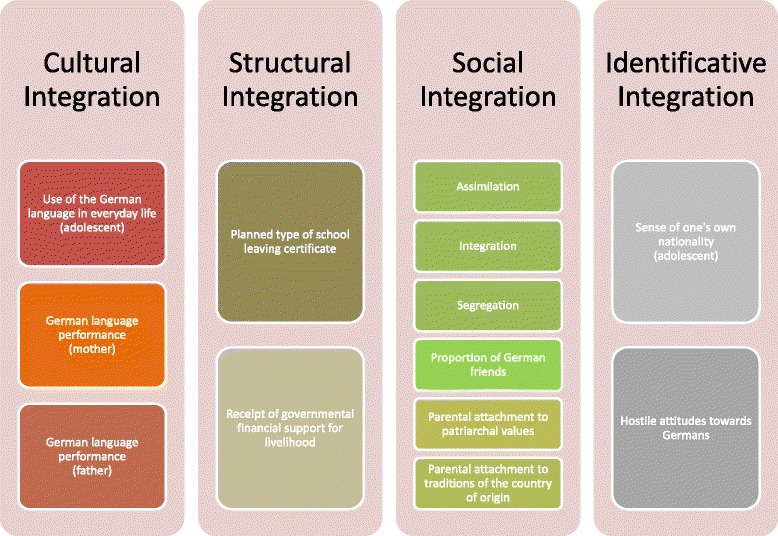
Operationalization of the four facets of integration according to Esser [2, 3]

According to Esser’s [14] theoretical framework, the concept of integration was split into four categories of integration, each operationalized individually according to the suggestions of the author [2, 3]—Fig. 2.

I) Cultural Integration

The use of German language in everyday life was measured with four items, for example: “In which language do you yourself mostly watch TV at home?” The answer categories were “German,” five other specific languages, and “other.” Participants were allowed to choose more than one language. The answers were dichotomized into German/German and other (1) and exclusively Non-German (0). A sum score was built across the four dichotomized items that ranged from 0 (speaking German in none of the everyday contexts) to 4 (speaking German in all of the everyday life contexts). The items were constructed by the Criminological Research Institute of Lower Saxony and used in previous representative studies in 2001 [11] and 2008 [15]. The adolescent rated his/her mother’s and/or father’s language performance according to the school grade rating system, which ranges from 1 to 6. For German school grades, 1 equals very good and 6 equals insufficient. This item was also constructed and used previously by the Criminological Research Institute of Lower Saxony [15].

II) Structural Integration

A single item with three answer categories was used to assess the planned type of school leaving certificate. In line with the German school system, it was possible to choose between a secondary general school certificate (9 years) = “Hauptschulabschluss,” a secondary modern school certificate (10 years) = “Realschulabschluss,” or a general qualification for university entrance/Hiqh school diploma (12 or 13 years) = “Abitur.” The item was constructed by the Criminological Research Institute of Lower Saxony and was previously published [16].

In order to assess whether the students received financial support from the government to support a secure existence, they were asked whether their parents or they themselves lived on social welfare (unemployment pays “Hartz IV” or welfare aid according to German social legislation). If they answered yes (versus no or I don’t know), the student received a “positive” welfare status score. The item was constructed by the Criminological Research Institute of Lower Saxony and was published previously [16].

III) Social Integration

**Fig. 3:**
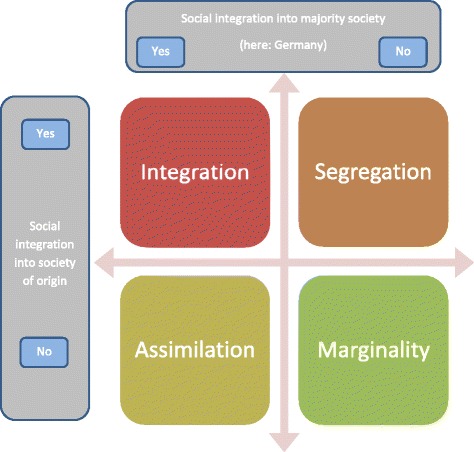
Categories of Social Integration

Social Integration was operationalized according to Esser’s [2, 3] theoretical framework. He distinguished four ways of including immigrants in the social system. They differ with respect to the extent to which the immigrant is included a) in the destination country and b) in the society of origin—see Fig. 3. Three of four variants of Social Integration were assessed: Integration, Assimilation, and Segregation. Integration refers to the immigrant’s orientation toward and social participation in both the original and destination societies and was measured via the single item: “People of my origin who live in Germany should maintain their own culture, but at the same time, they should also adapt to the German culture.” Assimilation describes the immigrant’s integration into the mainstream society along with a simultaneous dissociation from the society of origin and was assessed with the following item: “People of my origin who live in Germany should give up their own culture and adapt to the German way of life, thus think and act just like Germans.” The concept of Segregation describes the opposite of Assimilation and was measured with three items. An example item from the scale is “People of my origin who live in Germany should marry only amongst each other.”

This scale exhibited an acceptable internal consistency (Cronbach’s α = .75). Items were rated on a 4-point scale with the answer formats “not true,” “marginally true,” “rather true,” and “exactly true.” The described measure of acculturation has been used and published before [17] and was originally developed by Berry et al. [18].

Parental attitudes toward integration were measured with six items. An exploratory factor analysis revealed that the items were distributed between two factors. Thus, two scales, each with three items resulted: Scale 1—Parental attachment with patriarchal values, and Scale 2—Parental attachment with the traditions of the country of origin. The items were rated on a 4-point scale with the answer formats “not true,” “marginally true,” “rather true,” and “exactly true” and constructed by the Criminological Research Institute of Lower Saxony. They were used already in another representative study with adolescents [15]. Example items are: “My parents think that the man should be the head of the family” (Scale 1) and “My parents actively try to maintain the traditions of our country of origin” (Scale 2). The mean of the two scales was used in the multivariate analyses.

Esser [2, 3] considered interethnic friendships to be an additional indicator of social integration. This was operationalized by the proportion of German friends in the group of the participant’s five best friends.

IV) Identificative Integration

The sense of self concerning one’s own nationality was assessed with one item asking “How do you perceive yourself?” The answer categories were “German,” five specifically named other nationalities, and the category “other.” For analysis, the item was dichotomized into the categories “German” and “Non-German.” This item was previously constructed and tested in another study by the Criminological Research Institute of Lower Saxony [15].

German-hostile Attitudes were assessed with a nine-item scale. This scale, constituting a one-dimensional construct, was previously applied [15]. Items had to be answered on a 4-point scale with the answer formats “not true,” “marginally true,” “rather true,” and “exactly true” and were constructed by the Criminological Research Institute of Lower Saxony. An example item is: “Germans are less worthy than people of my origin.” The internal consistency of the scale was acceptable (Cronbach’s α = .87).

In addition, adolescents with an immigration background were asked how old they were when they came to Germany. The answer category “I was born in Germany” was offered here. They were also asked for the number of years their corporal parents had lived in Germany (as of the current date) (separately for the mother and father). The answer category “since their birth” was also offered. The item was constructed by the Criminological Research Institute of Lower Saxony and field-tested before [15].

### Statistical Analyses

We employed methods of descriptive and inference statistics to analyze the epidemiological parameters. The data analyses were implemented in SPSS 21. Differences in frequencies were assessed with Chi^2^ tests; differences in continuous variables were assessed with t tests. Levene’s tests to check the distribution of variances were carried out before the t tests, and if necessary, the *p*-value was adapted. Because of the sample size, the significance level in the epidemiological part of the results was set to *p* = .01. The proportion of missing data was small (below 5%) for the analyzed variables. Because of the sample size of the representative sample (*N* = 9512), missing data were not imputed for the epidemiological analysis. Tables 1 and 2 present the proportions of missing data. As a sensitivity analysis, the epidemiological analysis (research question 1) was also carried out separately for males and females in order to detect whether the potential different consumption behaviors of the immigrants and natives were based solely on sex.

To analyze research question 2, which asked whether immigration-specific variables could predict binge drinking in adolescents with an immigration background, the following was determined. The analyzed sample was a subsample and thus smaller (about 1/5 of the whole sample). Therefore, the level of significance in the regression analysis was set to *p* = .05. Furthermore, using only complete cases and not imputing missing values would have led to a further reduction in the subsample size, especially because different subjects had missing values on the different variables with single missing values. Thus, missing values were imputed with the mean of the scale/the mean of the continuous variable. This process resulted in a stable sample size for the regression of *N* = 1763. Categorical variables were imputed conservatively; for example, if the question of whether the family received welfare was missing, the item was imputed with “no” (0) because we wanted to avoid intentionally creating statistical differences when we were not sure about the information. A multiple logistic regression analysis with the dependent variable “binge drinking—yes/no” and 16 predictors was carried out. In the interpretation of the results, we took into account not only statistical significance but also ORs and their confidence intervals. The number of predictors did not conflict with the sample size because a rule of thumb suggested that the number of predictors should not exceed the square root of the sample size [19]. We also checked for multicollinearity in the potential predictors. The associations of the 16 predictor variables were low to moderate (*r* < .5), with the exception of a moderate correlation between “German-hostile attitudes” and “Segregation” (*r* = .510). The associations are depicted in detail in Additional file 1. None of the predictors were excluded from the analysis. As a sensitivity analysis, logistic regression analyses for detecting immigration-associated predictors of binge drinking were carried out for the two largest immigrant groups separately as a subgroup analysis. The subsample sizes (*N* = 490 for adolescents with an immigration background from the former Soviet Union; *N* = 354 for adolescents with a Turkish immigration background) was large enough to ensure that the number of predictors did not have to be reduced (√354 = 18.8 → 19 predictor variables possible). The goal of this sensitivity analysis was to determine whether immigration-specific predictors for binge drinking vary by the country of origin because the prevalence of binge drinking in immigrant adolescents varies considerably and depends on their roots.

## Results

### Epidemiology of the substance consumption of the whole sample and in comparison between participants with an immigration background versus no immigration background

#### Lifetime prevalence

The lifetime prevalence for use of the three examined substances alcohol, tobacco, and cannabis by 9th graders was highest for alcohol (84.6%), followed by tobacco (35.0%) and cannabis (12.9%).

There were significant differences in the lifetime prevalence of alcohol use between immigrants (74.7%) versus non-immigrants (89.7%) (*p* < .001) and in the lifetime prevalence of tobacco use between immigrants (40.1%) and non-immigrants (34.3%) (*p* < .001) as well as in the lifetime prevalence of cannabis use: for immigrants, it was 15.2%, and for non-immigrants, it was 12.6% (*p* = .002).

In summary, the following could be noted: At an average age of 15 years, compared with German adolescents without an immigration background, significantly fewer adolescents with an immigration background had already tried alcohol, but they were significantly more likely to report experience with cigarettes and cannabis.

#### Twelve-month prevalence

The detailed percentages of the 12-month prevalence for the consumption of alcohol, tobacco, and cannabis are shown in Table 2. In total, the percentage of regular consumers (once a week or more) was 11.3% for alcohol, 12.3% for tobacco, and 1.7% for cannabis.

**Table 2:** 12-month prevalence rate (%) for alcohol, tobacco, and cannabis use

	Alcohol		Tobacco		Cannabis	
	Frequency (*n*)	%	Frequency (*n*)	%	Frequency (*n*)	%
Never	1659	17.4	6366	66.9	8142	85.6
1 to 12 times a year	4934	51.9	1385	14.6	804	8.5
Several times a month	1655	17.4	347	3.7	144	1.5
Once/Several times a week	1037	10.9	404	4.3	128	1.3
Daily	39	0.4	763	8.0	39	0.4
Missing values	188	2.0	247	2.6	255	2.7
Total	9512	100.0	9512	100.0	9512	100.0

The 12-month prevalence for alcohol consumption differed significantly between immigrants and non-immigrants (Chi^2^(4) = 327.98; *p* < .001), most clearly in the percentages of the “never”-users (13.8% of non-immigrants compared with 30.2% of immigrants). On the other hand, the percentages of regular consumers (once a week or more) differed less on a descriptive level: 12.1% of non-immigrants versus 9.6% of immigrants.

There was a difference in the consumption of tobacco in the previous 12 months between immigrants and non-immigrants: The percentage of regular smokers was slightly higher for immigrants (14.5%) than for adolescents without an immigration background (12.0%) (Chi^2^(4) = 15.41; *p* = .004). The percentage of non-smokers (“never” in the previous 12 months) was at about 2/3 for both groups (69.6% for non-immigrants, 66.0% for immigrants).

**Fig. 4:**
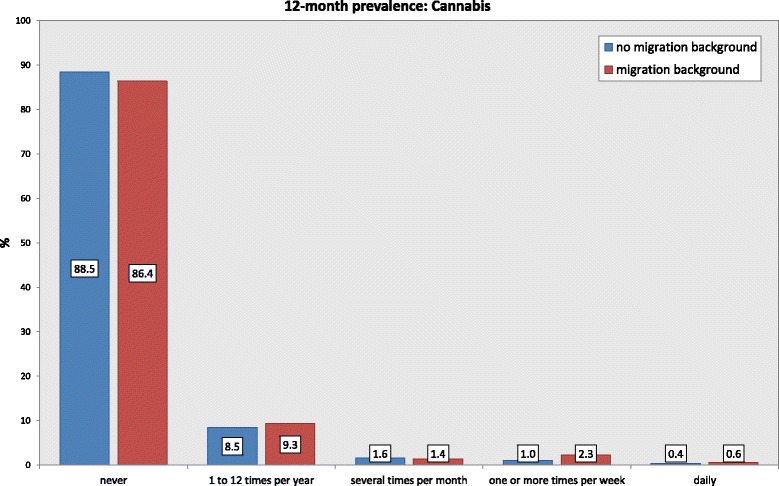
12-month prevalence rate for cannabis use according to immigration background

The consumption of the drug cannabis, which is illegal in Germany, was higher for immigrants than non-immigrants for the previous 12 months. Whereas 1.4% of the adolescents without an immigration background reported consuming cannabis regularly (at least once a week), the percentage of adolescents with an immigration background who reported this was 2.9%. The percentage of the “never” consumers was 88.5% for non-immigrants and 86.4% for immigrants. Statistically, these differences were significant: Chi^2^(4) = 26.51; *p* < .001. The 12-month prevalence for cannabis use in the group comparison is shown in Fig. 4.

#### Age at first consumption

The age at first consumption (for only the adolescents who reported a positive lifetime prevalence) was the lowest for alcohol (12.9 years), followed by tobacco (13.3 years), and cannabis (14.3 years). There are no significant differences in the age at first consumption between immigrants and non-immigrants when only the substance users (lifetime) were the focus. The means, standard deviations, and inferential statistics of the age at first consumption are depicted in Table 3.

**Table 3:** Age at first consumption of alcohol, tobacco, and cannabis—differentiated by immigration background

Substance		Total sample Mean (SD)	Total sample Median	Adolescents with immigration background Mean (SD)	Adolescents without immigration background Mean (SD)	*t* value	*p*-value	95 % confidence interval for the difference
Alcohol	(*N* = 7810)	12.87 (1.95)	13.00	12.75 (2.33)	12.90 (1.83)	2393	.017	.027–.271
Tobacco	(*N* = 3153)	13.28 (1.86)	14.00	13.13 (2.11)	13.33 (1.76)	2457	.014	.040–.357
Cannabis	(*N* = 1156)	14.33 (1.07)	14.00	13.13 (2.11)	14.33 (1.04)	−.493	.622	−.177–.108

Includes only cases with a “positive” lifetime prevalence

#### Binge drinking

The binge drinking item had to be answered only by adolescents who reported that they had consumed alcohol within the previous 30 days. Therefore, data on binge drinking were available from *N* = 7038 adolescents. In relation to the total sample (*N* = 9512), the percentage of the binge drinkers was 30.1% as all the adolescents who had not consumed any alcohol in the previous 30 days were classified as non-binge drinkers (Binge drinking rate for Complete Case Analysis: 31.5%). This is a conservative estimate as girls (and boys) were rated as binge drinkers only when they had consumed five or more glasses of alcohol on one occasion.

**Fig. 5:**
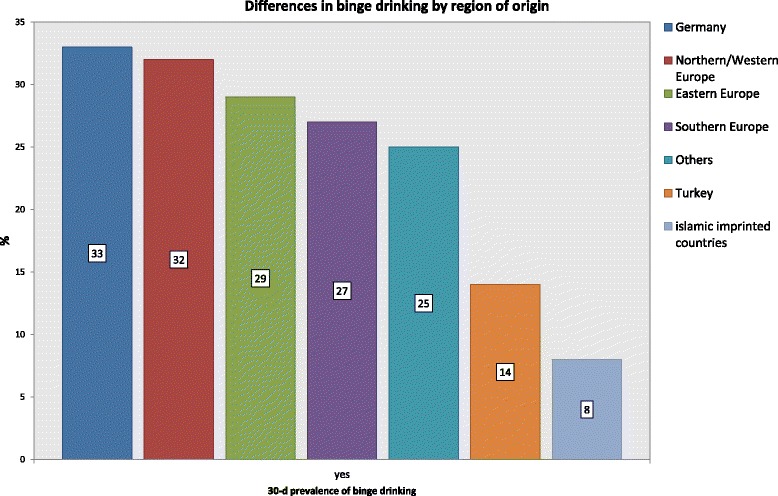
30-day prevalence rate for binge drinking by region of origin

In the following results the percentage of binge drinkers was always computed for the total sample. Adolescents with and without an immigration background showed a significant difference with respect to the prevalence of binge drinking: For adolescents with an immigration background, the percentage of binge drinking was 24.3%; for adolescents without an immigration background, it was 32.5% (Chi^2^(1) = 54.88; *p* < .001). This difference emerged with the same level of significance and in the same direction when only adolescents who had actually consumed any alcohol at all in the previous 30 days were considered (Chi^2^ (1) = 117.57; *p* < .001). The percentage of binge drinkers fluctuated significantly in the group of adolescents with an immigration background when country of origin was considered (Fig. 5). This analysis showed that adolescents with a Northern or Western European background exhibited drinking patterns that were similar to those of German adolescents, whereas adolescents from countries of origin where Islam is the prevailing religion were found to practice binge drinking significantly less often (Chi^2^(6) = 108.76; *p* < .001).

### Sensitivity Analysis

The epidemiological parameters lifetime prevalence, 12-month prevalence, and age at first consumption were additionally analyzed separately for boys and girls (with and without an immigration background). The goal was to detect whether the less harmful substance consumption patterns of adolescents with an immigration background reported in the literature were based on sex only (presumably girls). This hypothesis could not be confirmed. Both boys and girls with an immigration background showed less harmful alcohol consumption, i.e. lifetime prevalence (LTP), and their 12-month prevalence was significantly lower (*p* < .001) in comparison with boys and girls without an immigration background (boys LTP: 75.4 vs. 89.6%; girls LTP: 74.2 vs. 89.9%). Concerning tobacco and cannabis use, boys and girls with an immigration background showed potentially more harmful consumption behavior. The lifetime prevalence of tobacco use was significantly higher (*p* < .001) in girls with an immigration background (40.5%) in comparison with girls without an immigration background (32.9%). Furthermore, the lifetime prevalence of cannabis use was significantly higher (*p* < .001) in boys with an immigration background (19.5%) in comparison with boys without an immigration background (14.4%). This also accounted for the 12-month prevalence rates of both cannabis and tobacco use in boys. The rate of daily smokers was higher in boys (12.0%) as well as in girls (8.0%) with an immigration background in contrast to their “German” counterparts (7.9 and 7.4%, respectively). The age at first consumption was not significantly different, even though boys and girls with an immigration background had slightly lower ages at first consumption than boys and girls without an immigration background. The results are depicted in detail in Additional file 2.

### Immigration-specific predictors of binge drinking for adolescents with an immigration background

In a significant binary logistic regression model with *N* = 1763 adolescents with an immigration background (Chi^2^(17) = 110.372; *p* < .001), where 77.5% of the adolescent binge drinkers were correctly classified, we found the following: Binge drinking was positively associated with a targeted secondary general school certificate (9 years) (*p* < .001; OR: 2.92) or a secondary modern school certificate (10 years) (*p* < .001; OR: 2.15) (in contrast to the German high school diploma), the family not living on social welfare (*p* = .038; OR: 1.52), and attitudes favoring the assimilation of the adolescent him-/herself (*p* = .015; OR: 1.22) (see Table 4). For adolescents with an immigration background, binge drinking was negatively associated with personal attitudes that favored segregation (*p* < .001; OR: 0.64) as well as a stronger parental attachment to the traditions of the country of origin (*p* = .003; OR: 0.90) (see Table 4). The model explained 9.2% of the variance in binge drinking.

**Table 4:** Immigration-associated predictors for binge drinking in adolescents with an immigration background (*N* = 1763)

	Regression coefficient *β*	Standard error	Wald	df	*p*	OR	95 % confidence interval for OR	
							Lower value	Upper value
Years living in Germany (adolescent)	.042	.028	2.189	1	.139	1.043	.986	1.102
Years living in Germany (mother)	−.004	.007	.454	1	.501	.996	.983	1.008
Years living in Germany (father)	.002	.006	.071	1	.790	1.002	.990	1.013
Use of the German language in everyday life (adolescent)	.109	.072	2.278	1	.131	1.115	.968	1.286
German language performance (mother)	−.093	.059	2.474	1	.116	.911	.811	1.023
German language performance (father)	−.053	.055	.926	1	.336	.948	.851	1.057
Planned type of school leaving certificate [reference category = high school diploma (at least 12 years)]:			41.369	2	<.001			
Secondary general school certificate (9 years)	1.070	.197	29.509	1	<.001	2.915	1.982	4.289
Secondary modern school certificate (10 years)	.766	.136	31.640	1	<.001	2.151	1.647	2.808
Receipt of governmental financial support for livelihood (social welfare)^a^	.416	.201	4.301	1	.038	1.517	1.023	2.248
Assimilation	.195	.081	5.851	1	.016	1.216	1.038	1.424
Integration	.015	.054	.071	1	.789	1.015	.912	1.129
Segregation	−.444	.117	14.317	1	<.001	.641	.510	.807
Proportion of German friends	.028	.197	.021	1	.886	1.029	.699	1.514
Parental attachment to patriarchal values	.003	.039	.006	1	.937	1.003	.929	1.083
Parental attachment to traditions of the country of origin	−.101	.035	8.440	1	.004	.904	.844	.968
Sense of one’s own nationality (adolescent)^b^	.157	.143	1.205	1	.272	1.169	.884	1.546
German-hostile attitudes toward Germans	.030	.016	3.393	1	.065	1.031	.998	1.064
Constant	−2.166	.649	11.147	1	.001	.115		

^a^0 = yes; 1 = no

^b^0 = German; 1 = Non-German

### Sensitivity Analysis

The two binary logistic regression analyses for the subgroups of adolescents with a former Soviet Union or Turkish immigration background resulted in significant models (*p* = .042/*p* = .003, respectively) whose details are depicted in Additional file 3. As expected, the number of significant predictors was smaller in samples with *N* = 490/*N* = 354, respectively, as was found in the whole group of all adolescents with an immigration background. In conclusion, for adolescents with a former Soviet Union immigration background, only two variables predicted binge drinking (BD): the planned type of school leaving certificate (fewer years of education were associated with a higher risk of BD) and the number of years the mother had already been living in Germany (more years were associated with a lower risk of BD). For adolescents with a Turkish immigration background, only the planned type of school leaving certificate reached statistical significance in predicting binge drinking. However, for adolescents with Turkish roots, immigration-associated variables explained 18.9% (R^2^) of the variance, a finding that is twice as high as the amount of variance explained in the whole group of adolescents with an immigration background (9.2%) and also twice as high as in the other subgroup (8.0%).

## Discussion

### Epidemiology of substance consumption: total sample

As expected, regarding the lifetime prevalence rates for substance consumption, alcohol came in first place, followed by tobacco and the (in Germany) illegal drug cannabis [8, 20, 21]. The same order was also reflected for the age at first consumption, which increased across the three substances, respectively. Representative surveys in Germany, carried out by the BZgA (German Federal Centre for Health Education) [8, 20–22], and representative studies on a European base [13, 23] were considered for the interpretation of this study. The representative European ESPAD study, which also reported data that were analyzed separately for Germany, focused on exactly the same age group (15-year-olds) as the study carried out by us and was therefore very well-suited for use as a comparison group. However, the data from our study were more recent and allowed us to compute additional analyses because of the inclusion of possible risk and protective factors.

### Alcohol

In our sample, the 12-month prevalence rate for alcohol use (80.6%) was in a plausible range for average 15-year-olds. The BZgA reported rates of 46.0% for 12–15-year-olds and 88.9% for 16–17-year-olds in their samples [8]. The drastic rise from the age of 16 onwards can be explained by the fact that in Germany, adolescents can legally buy alcohol after they turn 16. In the European comparison, referring to the ESPAD study, which interviewed 15-year-olds, the 12-month prevalence rate for alcohol use was almost equal to the one in our study (80.6 versus 79.0% in the European average) [13]. In this Europe-wide study, German adolescents had an even higher 12-month prevalence rate of 89.0%.

Also the lifetime prevalence rate for alcohol use was within the expected range for the average age of the sample. The 84.6% found in this study ranked between the findings of 57.7% for 12–15-year-olds and the 92.5% for 16–17-year-olds that were published in the BzgA survey of German adolescents carried out in the year 2014 [8]. In the European ESPAD study, the lifetime prevalence rate was on average 87.0% for all participating countries; for Germany alone, it was 92% [13].

According to the European representative study [13] reported in three quarters of the participating countries, at least half of the students stated that they had drunk at least one glass of an alcoholic beverage at the age of 13 or younger. In our study, the average age at which alcohol was first consumed was 12.9 years. One German representative study reported 13.6 years as the age at which alcohol was first consumed for the assessment year 2011 in a sample of 12–17-year-olds and referring only to ever-users [22]. According to the authors, the age at first alcohol consumption moved from 13.0 years in 2004 to an average that was 6 months later in 2011.

For the adolescents we focused on in our study, the frequency of binge drinking (30.1%) was above the rate reported in the most recent German BzgA survey for 12–15-year-olds (5.6%) and below the rate of the frequency found in that survey for 16–17-year-olds (33.9%) [8]. As found in an earlier representative study with 15-year-olds, at this age, adolescents show a drinking pattern that resembles that of 16-year-olds more than that of 12-year-olds [9, 24]. The rate reported in our study lies below the rate of the European average: the ESPAD study reported 39.0% binge drinking (five drinks in a row) in the past 30 days [13].

### Tobacco

The lifetime prevalence rate for tobacco use found in our study of 35.0% was slightly higher than the one reported by the BZgA of 28.3%; however, the latter one involved 12–17-year olds [21]. Considering the age at first consumption being on average 13.3 years (in our study), a lower lifetime prevalence is to be expected when 12-year-olds are also explicitly included in the survey. For 2012, the BZgA [22] also reported a significantly higher age at first consumption (for 12–25-year-olds) of 14.4 years for participants with a positive lifetime prevalence. However, regarding the method, it has to be mentioned that statistically, the average age at first consumption will increase as the upper age limit of the random sample increases. For example, the BZgA reported [22] two different ages at first consumption—one for 12–17-year-olds and one for 12–25-year-olds, the latter one being significantly higher (corresponding to what is desired by society and politics). In our study of 9th graders, the percentage of those who reported smoking every day (8.2%) was higher than the figure reported in the representative survey for Germany, which looked at 12–17-year-olds: 4.6% [21].

The European representative studies reported rather “higher” frequencies than the ones we found [13]: The average lifetime prevalence rate for tobacco use was reported to be 54.0% for European 15-year-olds. For Germany, they found a lifetime prevalence rate of 61.0%. This is clearly above the rate of 35.0% found in our study. One explanation can be the changing smoking behavior in young adolescents, which has been seen in Germany. The cited European data were older, and thus a higher prevalence rate could be expected. The adolescents who had already tried tobacco reported the age at first consumption as 13.3 years in our study. This seems to be in accordance with the European results where 31.0% of all those interviewed had already tried a cigarette at the age of 13 or younger; in Germany, the rate was 33.0%.

### Cannabis

In the latest European Drug Report dated 2014 [23], the lifetime prevalence rate for cannabis was listed at 24.0% for the total group of 15–16-year-olds and explicitly for Germany at 19.0%. According to the ESPAD study, the European average for the lifetime prevalence rate for cannabis use is 17% [13]. This is remarkably higher than in the slightly younger sample of 9th graders (12.9%) that we had at hand. In a representative German study from 2014 [20], the lifetime prevalence rate for cannabis use for 12–17-year-olds was reported as 7.8%. This is below the rate of 12.9% that we found.

The 12-month prevalence rate for the use of cannabis for 15–16-year-olds is quoted as 20% in the European Drug Report [23]. This source reports the 12-month prevalence rate for Germany for adolescents and young adults as 11.1%. This parameter is very close to the 11.7% reported in the current study. The figures reported in the ESPAD study were also in a similar range: 13% for the average European 12-month prevalence rate for cannabis use (15% for Germany) [13]. The German study by the BZgA [20] listed a lower 12-month prevalence rate for cannabis use for 12–17-year-olds: 5.6%.

Compared with the data from the German BZgA survey, the age at first consumption of cannabis in our study was lower (14.3 years compared with 16.7 years) [22]. However, this can again be partially explained by the upper age limits that were chosen for the samples (see above). The BZgA included “ever”-users from the age range of 12 to 25 in the definition for the age at first consumption, whereas in our study, only 9th graders were assessed.

### Epidemiology of substance consumption: differences between immigrants and non-immigrants

In our sample, and as had already been reported in the literature [8, 9], a lower percentage of immigrants reported drinking any alcohol at all (lower lifetime prevalence, lower 12-month prevalence). The immigrant sample also reported engaging less in binge drinking, even though the age at first consumption for alcohol did not differ significantly between immigrants and non-immigrants.

However, it is interesting that adolescents with an immigration background reported smoking and consuming cannabis more often (according to the 12-month prevalence rate). Also, the percentage of those who had ever tried the two substances (lifetime prevalence) was higher for immigrants. Gender-specific analyses also provided statistically significant support for these results.

This means that the culturally based rejection of alcohol might not generally have a protective effect on substance consumption, but it is possible that the behaviors of trying and consuming have simply been shifted to other legal or illegal substances.

### Immigration-specific protection and risk factors for binge drinking for adolescents with an immigration background

In the sample of students with an immigration background, variables related to immigration explained almost 10% of the variance in binge drinking. However, this is not surprising because, as shown in preliminary studies by others and ourselves (e.g. [16, 25, 26]), a number of other factors that do not have anything to do with immigration background are obviously related to binge drinking for these adolescents. Such factors were not included here as the focus was solely on predictors related to the concept of immigration.

It is interesting that the variables that were used to operationalize cultural integration (e.g. use of German language) and identificative integration (e.g. sense of one’s own nationality) did not show any relation to the behavior of binge drinking. The length of time the adolescent him-/herself or his/her parents had been living in Germany also did not predict binge drinking.

Contrary to the above findings, both variables of structural integration (planned type of school leaving certificate and living on social welfare) showed a significant connection to binge drinking. In this context, living on social welfare acted as a protective factor for binge drinking as adolescents whose parents did not depend on state support showed a 1.5 (OR: 1.52) times higher risk of engaging in binge drinking. This result had already been found in another representative study of 45,000 German adolescents [16] in exactly the same age group. Furthermore, comparable to our study, in their sample of 11,000 students in England in the same age group, Bellis et al. [27] found that children with greater expendable incomes reported more unsupervised, frequent, and heavy drinking. This was also found in a Spanish adolescent sample [28]. The most obvious explanation for this effect may be that binge drinking requires financial expenditures that adolescents with fewer financial resources cannot easily afford.

We found that the planned type of school leaving certificate acts protectively as long as it is the highest achievable school leaving certificate available in Germany: the “Abitur” (University entrance diploma). The planned types of school leaving certificates that go together with a lower number of school years (secondary general school certificate: 9 years, secondary modern school certificate: 10 years) were associated with a higher likelihood of engaging in binge drinking (OR: 2.92 for secondary general school; OR: 2.15 for secondary modern school). This is a very stable result that was also confirmed in the origin-specific subgroup analysis of adolescents with immigration backgrounds from Turkey or the former Soviet Union. It can be assumed that the higher the planned type of school leaving certificate is, the higher the structural integration will be even though the adoption of low educational expectations may reflect integration into sectors of German society that have low educational expectations. However, in Germany, the Abitur is regarded as the school leaving certificate with the highest esteem from parents and students (e.g. [29]). Although in another representative study in Germany with 15-year-old adolescents, the planned type of school leaving certificate—independent of students’ immigration background—was not related to the frequency of their binge drinking [16], other studies have offered results that are in line with the results found in our study. For example, the BZgA, who regularly analyze the consumption behavior of adolescents and young adults in Germany, also reported that for the 5000 adolescents they analyzed, the prevalence rate for binge drinking increased when planned school leaving certificates were lower [8]. Also, a US study found that higher educational commitment was correlated with less frequent drinking and with drinking smaller amounts [30]. It seems that in our study, in the sample of adolescents with an immigration background, aiming at a higher type of school leaving certificate acted as a protective factor for substance consumption in the sense of binge drinking. It is also a known fact that binge drinking is associated with lower academic success—which, in Germany, is reflected in the (obligatory) choice of school type on the basis of the achievements of the first 4 years of elementary school [31, 32].

Several variables from the area of social integration were found to act as significant predictors of binge drinking for adolescents with an immigration background. For example, as expected, assimilation—meaning giving up the culture of one’s country of origin and concentrating on the German culture—was found to be a risk factor (OR 1.22). Such adolescents adapted to the drinking habits of their German friends who did not have an immigration background and whose prevalence of binge drinking was higher [8]. This shows that assimilation, which is often favored by the majority groups in a society [33] and is accepted and practiced by highly educated young people [34], can also have negative effects.

In contrast to this, segregation, which is normally considered critical from a social-cultural perspective, acts as a protective factor when it comes to critical alcohol consumption. Adolescents who strongly orient themselves toward the culture of their countries of origin and at the same time refuse the German culture have a lower risk of becoming binge drinkers (OR: 0.64). The cultural orientation of the parents has an effect in the same direction: Adolescents whose parents remain strongly attached to the traditions of their countries of origin exhibit a lower risk for binge drinking (OR: 0.90). Of course, segregation has other negative results for a society (see e.g. [35]); however, such effects lie outside the scope of this study. Integration (versus segregation and versus assimilation), which is often regarded as the ideal form of social integration (e.g. [36]) from a political point of view, does not have either protective or negative relations to substance consumption in the context of binge drinking.

The role of parental substance consumption behavior in the consumption patterns of adolescents is well-studied. Parental substance abuse raises the risk for adolescent substance abuse [37]. Furthermore, parental substance-related attitudes that do not explicitly deny risky substance consumption are positively associated with higher/more harmful adolescent consumption behavior [38]. We showed that parents’ substance-unspecific attitudes—and in this case attitudes toward the culture of the immigrant and the country of origin—can also predict risky substance consumption in adolescents.

For adolescents originating from the former Soviet Union, only one additional variable had a small protective effect: the longer the mother had been living in Germany, the smaller the risk for binge drinking for the adolescent. Here, subtle processes of adapting to the new culture as described in the introduction may play a role. For adolescents with a Turkish immigration background, the intensity of their religious beliefs and activities surely plays a role in predicting their binge drinking. As we published previously [16], being religiously active oneself and being integrated into a religious community are factors that protect against binge drinking. This holds especially for Turkish adolescents living as Muslims.

### Critical reflection on the study

The study represents a large and representative sample of 9512 adolescents. It is representative for one German state rather than for Germany as a whole. However, all related legal regulations regarding the legal age for the purchase of substances, youth protection laws, the legalization of cannabis, and taxes on substances are nationwide regulations and are therefore the same for all German states. The definition of binge drinking used in this study (five drinks on one occasion for all adolescents) is so far the one with the longest tradition used in research and is a more liberal definition. Since 2012, in some studies, the stricter definition of four standard glasses on one occasion is used for girls for binge drinking. For this reason, the figures reported in this study must be viewed as conservative. Using the stricter definition would result in higher prevalence rates for binge drinking for girls and on average for the whole sample.

However, another restriction that applies to all epidemiological studies that collect data on substance consumption via self-disclosure is the potential for bias due to (a lack of) social acceptability. It is primarily information on the consumption of illegal substances that can be influenced by this, and thus rates might actually be higher in reality. However, the bias in this present study can be assumed to have the same scope of influence as found in other studies with adolescents and young adults. Therefore, the prevalence rates from representative studies are comparable even though the absolute figures might be higher in reality.

The method that we used to define immigration background differs from that used in other studies, particularly in the US. We did not differentiate between first-generation immigrants and descendants of immigrants living in the immigration country for a second generation. The latter situation applied to the majority of the current sample. Thus, the present results should be interpreted as accounting for second-generation immigrants.

It was not the goal of the current analysis of predictors of binge drinking to provide a comprehensive picture of the protective and risk factors for binge drinking in general. From our point of view, there are already a sufficient number studies that have done this (e.g. [26, 39–41]). By contrast, the current study focused exclusively on analyzing the subgroup of adolescents with an immigration background to shed light on the phenomenon that “adolescents with an immigration background drink less,” which has been reported in the literature. The goal was to reveal immigration-specific protective and risk factors to make the acquired knowledge available for target-group-specific prevention measures. However, more research is needed to specify the indicators and predictors of binge drinking in adolescents with immigration backgrounds (e.g. expected educational certificate).

## Conclusions

Adolescents with an immigration background (with respect to “second-generation immigrants”) do indeed drink less but under the premise of not yet being assimilated into the “new” society as they tend to favor segregation and are financially dependent on social welfare. As a lack of integration has a far graver effect on a society, the task is to influence patterns of alcohol consumption and counteract its negative development as soon as possible with target-group-oriented prevention measures while simultaneously supporting integration into the society of the immigration country as it unfolds. The assessed prevalence rates of tobacco and cannabis consumption, which presumably result in negative effects on the health of adolescents with an immigration background, show that prevention is necessary for these groups.

## Additional files

Additional file 1: Analysis of multicollinearity. (DOCX 23 kb)
Additional file 2: Gender-specific epidemiology of substance consumption: Differences according to immigration background. (DOCX 17 kb)
Additional file 3: Subgroup-specific analysis for immigration-associated predictors of binge drinking for the two largest migrant groups. (DOCX 30 kb)
