# Supplementary material for: Substance consumption in adolescents with and without an immigration background: a representative study—What part of an immigration background is protective against binge drinking?
Source: BMC Public Health. 2016 Nov 14;16:1157. doi: 10.1186/s12889-016-3796-0 (PMC5109665; doi:10.1186/s12889-016-3796-0)
Supplement: Additional file 1: — Analysis of multicollinearity. (DOCX 23 kb) [file 12889_2016_3796_MOESM1_ESM.docx]

**Pearson correlation coefficient of potential predictors**

|  | **1** | **2** | **3** | **4** | **5** | **6** | **7** | **8** | **9** | **10** | **11** | **12** | **13** | **14** | **15** |
| --- | --- | --- | --- | --- | --- | --- | --- | --- | --- | --- | --- | --- | --- | --- | --- |
| 1 Planned type of school leaving certificate | 1 | -0.076 | .043 | .103^**^ | -.154^**^ | -.138^**^ | .111^**^ | -.092^**^ | .035 | -.043 | -.145^**^ | -.164^**^ | -.105^**^ | .118^**^ | -.110^**^ |
| 2 Years living in Germany (adolescent) | -.076^**^ | 1 | .294^**^ | .175^**^ | -.165^**^ | -.111^**^ | .118^**^ | -.010 | -.001 | -.026 | .056^*^ | .035 | -.032 | .077^**^ | .051 |
| 3 Years living in Germany (mother) | .043 | .294^**^ | 1 | .436^**^ | -.435^**^ | -.152^**^ | .198^**^ | .026 | .031 | .074^**^ | -.080^**^ | -.077^**^ | -.193^**^ | .140^**^ | -.085^**^ |
| 4 Years living in Germany (father) | .103^**^ | .175^**^ | .436^**^ | 1 | -.208^**^ | -.380^**^ | .161^**^ | -.041 | -.023 | .075^**^ | -.058^*^ | -.095^**^ | -.144^**^ | .137^**^ | -.053^*^ |
| 5 German language performance (mother) | -.154^**^ | -.165^**^ | -.435^**^ | -.208^**^ | 1 | .336^**^ | -.327^**^ | .129^**^ | .007 | -.021 | .233^**^ | .134^**^ | .374^**^ | -.228^**^ | .223^**^ |
| 6 German language performance (father) | -.138^**^ | -.111^**^ | -.152^**^ | -.380^**^ | .336^**^ | 1 | -.199^**^ | .171^**^ | .076^**^ | -.013 | .162^**^ | .116^**^ | .245^**^ | -.209^**^ | .146^**^ |
| 7 Use of German language in everyday life (adolescent) | .111^**^ | .118^**^ | .198^**^ | .161^**^ | -.327^**^ | -.199^**^ | 1 | -.059^*^ | -.046 | .043 | -.248^**^ | -.173^**^ | -.313^**^ | .337^**^ | -.240^**^ |
| 8 Receipt of social welfare **^1^** | -.092^**^ | -.010 | .026 | -.041 | .129^**^ | .171^**^ | -.059^*^ | 1 | .070^*^ | .002 | .092^**^ | .067^*^ | .118^**^ | -.050 | .106^**^ |
| 9 Integration | .035 | -.001 | .031 | -.023 | .007 | .076^**^ | -.046 | .070^*^ | 1 | -.006 | .204^**^ | -.073^**^ | .163^**^ | -.084^**^ | .086^**^ |
| 10 Assimilation | -.043 | -.026 | .074^**^ | .075^**^ | -.021 | -.013 | .043 | .002 | -.006 | 1 | .098^**^ | .015 | -.153^**^ | .150^**^ | .003 |
| 11 Segregation | -.145^**^ | .056^*^ | -.080^**^ | -.058^*^ | .233^**^ | .162^**^ | -.248^**^ | .092^**^ | .204^**^ | .098^**^ | 1 | .268^**^ | .458^**^ | -.338^**^ | .510^**^ |
| 12 Parental attachment with patriarchic values | -.164^**^ | .035 | -.077^**^ | -.095^**^ | .134^**^ | .116^**^ | -.173^**^ | .067^*^ | -.073^**^ | .015 | .268^**^ | 1 | .340^**^ | -.166^**^ | .353^**^ |
| 13 Parental attachment with traditions of the country of origin | -.105^**^ | -.032 | -.193^**^ | -.144^**^ | .374^**^ | .245^**^ | -.313^**^ | .118^**^ | .163^**^ | -.153^**^ | .458^**^ | .340^**^ | 1 | -.472^**^ | .435^**^ |
| 14 Sense of own nationality (adolescent) **^2^** | .118^**^ | .077^**^ | .140^**^ | .137^**^ | -.228^**^ | -.209^**^ | .337^**^ | -.050 | -.084^**^ | .150^**^ | -.338^**^ | -.166^**^ | -.472^**^ | 1 | -.359^**^ |
| 15 German hostile attitudes | -.110^**^ | .051 | -.085^**^ | -.053^*^ | .223^**^ | .146^**^ | -.240^**^ | .106^**^ | .086^**^ | .003 | .510^**^ | .353^**^ | .435^**^ | -.359^**^ | 1 |
| Legend: **^1^** Receipt of governmental financial support for livelihood 0 = yes; 1 = no; **^2^**0 = German; 1 = Non-German; p < .01. *p < .05. ** p < .01 | | | | | | | | | | | | | | | |
