# Supplementary material for: Substance consumption in adolescents with and without an immigration background: a representative study—What part of an immigration background is protective against binge drinking?
Source: BMC Public Health. 2016 Nov 14;16:1157. doi: 10.1186/s12889-016-3796-0 (PMC5109665; doi:10.1186/s12889-016-3796-0)
Supplement: Additional file 2: — Gender-specific epidemiology of substance consumption: Differences according to immigration background. (DOCX 17 kb) [file 12889_2016_3796_MOESM2_ESM.docx]

**Gender-specific epidemiology of substance consumption: Differences according to migration background**

Lifetime prevalence (%)

|  | male adolescents | | | female adolescents | | |
| --- | --- | --- | --- | --- | --- | --- |
|  | without migration background | with migration background | p-value | without migration background | with migration background | p-value |
| Alcohol | 89.6 | 75.4 | <.001 | 89.9 | 74.2 | <.001 |
| Tobacco | 35.8 | 39.8 | .017 | 32.9 | 40.5 | <.001 |
| Cannabis | 14.4 | 19.5 | <.001 | 10.7 | 11.2 | .643 |

12-month prevalence (%)

|  |  | male adolescents | | | female adolescents | | |
| --- | --- | --- | --- | --- | --- | --- | --- |
|  |  | without migration background | with migration background | p-value | without migration background | with migration background | p-value |
| Alcohol | Never | 13.6 | 29.3 | <.001 | 13.9 | 30.8 | <.001 |
|  | Several times/month | 21.5 | 13.6 |  | 17.1 | 12.3 |  |
|  | At least once/week | 16.4 | 13.3 |  | 6.7 | 5.5 |  |
|  | Daily | 0.6 | 0.6 |  | 0.2 | 0.3 |  |
| Tobacco | Never | 68.7 | 66.9 | .001 | 70.6 | 65.1 | .010 |
|  | Several times/month | 4.1 | 2.9 |  | 3.4 | 4.4 |  |
|  | At least once/week | 4.3 | 4.1 |  | 4.2 | 5.1 |  |
|  | Daily | 7.9 | 12.0 |  | 7.4 | 8.0 |  |
| Cannabis | Never | 86.7 | 82.6 | <.001 | 90.4 | 89.9 | .981 |
|  | Several times/month | 2.1 | 1.6 |  | 1.1 | 1.1 |  |
|  | At least once/week | 1.4 | 4.0 |  | 0.8 | 0.8 |  |
|  | Daily | 0.4 | 0.9 |  | 0.4 | 0.4 |  |

Age at first consumption

|  | male adolescents | | | female adolescents | | |
| --- | --- | --- | --- | --- | --- | --- |
|  | without migration background M (SD) | with migration background M (SD) | p-value T-Test | without migration background M (SD) | with migration background M (SD) | p-value T-Test |
| Alcohol | 12.86 (1.91) | 12.71 (2.46) | .103 | 12.95 (1.75) | 12.80 (2.20) | .065 |
| Tobacco | 13.28 (1.94) | 13.03 (2.38) | .058 | 13.39 (1.53) | 13.22 (1.85) | .081 |
| Cannabis | 14.33 (1.08) | 14.36 (1.24) | .762 | 14.31 (0.96) | 14.35 (0.95) | .698 |
