# Supplementary material for: Substance consumption in adolescents with and without an immigration background: a representative study—What part of an immigration background is protective against binge drinking?
Source: BMC Public Health. 2016 Nov 14;16:1157. doi: 10.1186/s12889-016-3796-0 (PMC5109665; doi:10.1186/s12889-016-3796-0)
Supplement: Additional file 3: — Subgroup-specific analysis for immigration-associated predictors of binge drinking for the two largest migrant groups. (DOCX 30 kb) [file 12889_2016_3796_MOESM3_ESM.docx]

**Subgroup-specific analysis for migration-associated predictors of Binge Drinking for the two largest migrant groups**

1. Adolescents with migration background from countries of the former Soviet Union (N = 490)

|  | **Regression coefficient β** | **Standard error** | **Wald** | **df** | **p** | **OR** | **95% confidence interval for OR** | |
| --- | --- | --- | --- | --- | --- | --- | --- | --- |
|  |  |  |  |  |  |  | Lower value | Upper value |
| Years living in Germany (adolescent) | .089 | .059 | 2.296 | 1 | .130 | 1.094 | .974 | 1.228 |
| Years living in Germany (mother) | -.046 | .021 | 4.528 | 1 | **.033** | .955 | .916 | .996 |
| Years living in Germany (father) | .013 | .016 | .682 | 1 | .409 | 1.013 | .982 | 1.045 |
| Use of the German language in everyday life (adolescent) | .160 | .128 | 1.562 | 1 | .211 | 1.174 | .913 | 1.509 |
| German language performance (mother) | -.039 | .115 | .116 | 1 | .734 | .962 | .768 | 1.204 |
| German language performance (father) | -.115 | .109 | 1.119 | 1 | .290 | .892 | .721 | 1.103 |
| Planned type of school leaving certificate [reference category = high school diploma (at least 12 years)]: |  |  | 8.940 | 2 | **.011** |  |  |  |
| General secondary school certificate (9 years) | .956 | .351 | 7.431 | 1 | **.006** | 2.601 | 1.308 | 5.171 |
| Secondary modern school certificate (10 years) | .603 | .247 | 5.969 | 1 | **.015** | 1.827 | 1.127 | 2.964 |
| Receipt of governmental financial support for livelihood (social welfare)* | .518 | .386 | 1.802 | 1 | .179 | 1.679 | .788 | 3.575 |
| Assimilation | .113 | .162 | .487 | 1 | .485 | 1.120 | .815 | 1.539 |
| Integration | .025 | .099 | .064 | 1 | .800 | 1.025 | .844 | 1.245 |
| Segregation | .038 | .218 | .031 | 1 | .861 | 1.039 | .678 | 1.592 |
| Proportion of German friends | -.022 | .342 | .004 | 1 | .949 | .978 | .500 | 1.914 |
| Parental attachment with patriarchal values | -.060 | .070 | .744 | 1 | .388 | .941 | .821 | 1.080 |
| Parental attachment with traditions of the country of origin | .025 | .063 | .157 | 1 | .692 | 1.025 | .907 | 1.159 |
| Sense of own nationality (adolescent)^#^ | .437 | .253 | 2.972 | 1 | .085 | 1.547 | .942 | 2.542 |
| German hostile attitudes | .018 | .030 | .376 | 1 | .540 | 1.019 | .960 | 1.080 |
| Constant | -3.216 | 1.234 | 6.791 | 1 | .009 | .040 |  |  |

Legend: *0 = yes; 1 = no ^#^ 0 = German; 1 = Non-German

1. Adolescents with migration background from Turkey (N = 354)

|  | **Regression coefficient β** | **Standard error** | **Wald** | **df** | **p** | **OR** | **95% confidence interval for OR** | |
| --- | --- | --- | --- | --- | --- | --- | --- | --- |
|  |  |  |  |  |  |  | Lower value | Upper value |
| Years living in Germany (adolescent) | .189 | .191 | .981 | 1 | .322 | 1.208 | .831 | 1.755 |
| Years living in Germany (mother) | .018 | .021 | .750 | 1 | .387 | 1.018 | .977 | 1.061 |
| Years living in Germany (father) | -.036 | .021 | 2.915 | 1 | .088 | .965 | .926 | 1.005 |
| Use of the German language in everyday life (adolescent) | -.020 | .182 | .012 | 1 | .912 | .980 | .686 | 1.400 |
| German language performance (mother) | -.145 | .167 | .752 | 1 | .386 | .865 | .624 | 1.200 |
| German language performance (father) | -.287 | .166 | 3.006 | 1 | .083 | .751 | .543 | 1.038 |
| Planned type of school leaving certificate [reference category = high school diploma (at least 12 years)]: |  |  | 7.289 | 2 | **.026** |  |  |  |
| Secondary general school certificate (9 years) | .668 | .715 | .873 | 1 | .350 | 1.950 | .480 | 7.915 |
| Secondary modern school certificate (10 years) | 1.347 | .529 | 6.489 | 1 | **.011** | 3.846 | 1.364 | 10.841 |
| Receipt of governmental financial support for livelihood (social welfare) * | .501 | .504 | .990 | 1 | .320 | 1.651 | .615 | 4.431 |
| Assimilation | .264 | .228 | 1.344 | 1 | .246 | 1.302 | .833 | 2.036 |
| Integration | -.048 | .167 | .084 | 1 | .772 | .953 | .687 | 1.322 |
| Segregation | -.595 | .333 | 3.189 | 1 | .074 | .551 | .287 | 1.060 |
| Proportion of German friends | -.309 | .584 | .280 | 1 | .597 | .734 | .234 | 2.305 |
| Parental attachment with patriarchal values | -.002 | .118 | .000 | 1 | .988 | .998 | .793 | 1.257 |
| Parental attachment with traditions of the country of origin | -.171 | .099 | 2.960 | 1 | .085 | .843 | .694 | 1.024 |
| Sense of own nationality (adolescent) ^#^ | -.354 | .436 | .660 | 1 | .416 | .702 | .298 | 1.650 |
| German hostile attitudes | .054 | .042 | 1.697 | 1 | .193 | 1.056 | .973 | 1.146 |
| Constant | -2.986 | 3.226 | .857 | 1 | .355 | .050 |  |  |

Legend: *0 = yes; 1 = no ^#^ 0 = German; 1 = Non-German
